# Unplanned urbanization and health risks of Dhaka City in Bangladesh: uncovering the associations between urban environment and public health

**DOI:** 10.3389/fpubh.2023.1269362

**Published:** 2023-10-19

**Authors:** Mohammad Anisur Rahaman, Abul Kalam, Md. Al-Mamun

**Affiliations:** ^1^College of Public Administration, Zhejiang University, Hangzhou, China; ^2^Department of Sociology, Bangabandhu Sheikh Mujibur Rahman Science and Technology University, Gopalganj, Bangladesh

**Keywords:** unplanned urbanization, urban environment, health risks, Dhaka city, public health

## Abstract

**Background:**

Dhaka City, the capital of Bangladesh, has experienced rapid and unplanned urbanization over the past few decades. This process has brought significant challenges to public health as the urban environment has become a breeding ground for various health risks. Understanding the associations between unplanned urbanization, the urban environment, and public health in Dhaka City is crucial for developing effective interventions and policies.

**Objectives:**

This review paper aims to uncover the associations between unplanned urbanization and health risks in Dhaka City, with a specific focus on the urban environment and its impact on public health. The objectives of this study are to examine the health challenges faced by the city’s population, explore the specific urban environmental factors contributing to health risks, analyze the socioeconomic determinants of health in unplanned urban areas, evaluate existing policies and governance structures, identify research and data gaps, and provide recommendations for future interventions.

**Methods:**

A comprehensive literature review was conducted to gather relevant studies, articles, reports, and policy documents related to unplanned urbanization, the urban environment, and public health in Dhaka City. Various databases and online resources were searched, and the selected literature was critically analyzed to extract key findings and insights.

**Results:**

The findings reveal that unplanned urbanization in Dhaka City has led to a range of public health risks, including air pollution, inadequate water and sanitation, poor waste management, overcrowding, slums, and substandard housing conditions. These environmental factors are strongly associated with respiratory diseases, waterborne illnesses, and other adverse health outcomes. Socioeconomic determinants such as poverty, income inequality, and limited access to healthcare further exacerbate the health risks faced by the urban population.

**Conclusion:**

Unplanned urbanization in Dhaka City has significant implications for public health. Addressing the associations between unplanned urbanization, the urban environment, and public health requires comprehensive policies and interventions. Improved urban planning, enhanced infrastructure, and better policy governance are essential for mitigating health risks. Furthermore, addressing socioeconomic disparities and ensuring equitable access to healthcare services are crucial components of effective interventions.

## 1. Introduction

Urbanization has long been closely associated with human development and progress, Dhaka City, the capital of Bangladesh, has experienced unprecedented urbanization in recent decades, leading to numerous health risks and environmental challenges (1). As the population continues to swell and urban infrastructure struggles to keep pace, the adverse impacts on public health are becoming increasingly evident. Uncontrolled and haphazard growth has left many people in cities without access to basic municipal services. Underprivileged infrastructure planning, lack of infrastructure, environmental degradation, traffic congestion, and housing shortage are among the most pressing challenges faced by Asian cities and towns in their pursuit of sustainable development (2). Due to fast urbanization, expanding population, and rapid industry, Bangladesh is experiencing environmental deterioration. The situation is exacerbated by the unplanned growth of cities, the unregulated silt load from buildings, and the big road works involving huge excavations during the rainy season. Between 1991 and 2019, Dhaka has experienced an average annual urban growth rate of 8%, with its outskirts expanding at an alarming rate of 43% during the same period. Specifically, the built-up areas in Dhaka’s outskirts expanded by 234 km^2^, surpassing the city’s growth of approximately 116 km^2^ between the same time (3, 4). This main objective of this research paper to provide a comprehensive analysis of the associations between unplanned urbanization, the urban environment, and public health in Dhaka City.

Urbanization without proper planning is a major issue worldwide, especially in the fast-growing megalopolises of developing nations. Dhaka City’s people face several threats to their health as a direct result of the city’s rapid and unchecked growth. (5). The Dhaka city monitors air quality based on five criteria pollutants, including Particulate Matter (PM10 and PM2.5), NO2, CO, SO2, and ozone. Air pollution has recently been a major issue (2, 6, 7) due mostly to industrial emissions, vehicle traffic, and the incineration of solid waste. Both asthma and chronic obstructive pulmonary disease (COPD) are linked to high levels of particulate matter and pollutants, including nitrogen dioxide (NO2) and sulfur dioxide (SO2) (7, 8). The increasing number of cases of respiratory illness in urban areas highlights the critical importance of taking immediate action to reduce air pollution and enhance air quality. Several climate-induced hazards, such as temperature swings, heavy and unpredictable rainfall, waterlogging, and flooding, have also been experienced in the country and have harmed urban life and livelihoods (9–12). However, climate change has been amplifying preexisting forces of devastation, giving rise to novel patterns of human migration, heightening preexisting venality, and fueling a surge of rapid and disordered urbanization (13). When climate change is compounded by issues like population growth, poverty, rural–urban movement, lack of education, unplanned urbanization, inefficient use of resources, and inadequate infrastructure (14, 15), the situation becomes dire.

Meanwhile, socioeconomic factors also play a significant role in shaping the health outcomes of urban residents. Dhaka City is characterized by high levels of poverty and income inequality, which exacerbate the health risks associated with unplanned urbanization (16). Limited access to healthcare services, inadequate nutrition, and substandard living conditions contribute to adverse health outcomes among marginalized populations (17). In addition to air pollution, inadequate water and sanitation infrastructure pose significant health risks in unplanned urban areas of Dhaka City. Contaminated water sources and inadequate sanitation facilities contribute to the spread of waterborne diseases, including diarrheal illnesses (1, 13). Furthermore, improper waste management practices and the lack of effective solid waste disposal systems have resulted in environmental contamination and the transmission of vector-borne diseases (18, 19). These challenges highlight the critical importance of addressing water and sanitation issues to protect public health in urban areas. The disparities in health outcomes further underscore the need for addressing social determinants of health and promoting equitable access to healthcare services in urban areas.

The Bangladesh Bureau of Statistics (BBS) reported that more than 18 lakh people, or almost 20% of the total population, reside in slums (20). Over 8.84 million people call the slums of Dhaka Division their home. On the other hand, a total of 40 lakhs (20, 21) is the estimate provided by several non-governmental groups. The appalling living conditions in the slums pose serious threats to the physical and mental health of the majority of the inhabitants. Except in extreme cases, they tend to avoid medical attention. They rely instead on the drugstore around the corner. Residents of slums are particularly vulnerable because of improper garbage management (21). Because of their low elevation, slums often become dumping grounds for trash from neighboring communities. The indiscriminate nature of slum garbage disposal is a leading cause of illness among slum residents. A Dhaka Calling Project study found that 34% of slum residents are afflicted by various infectious ailments due to the filthy atmosphere. The study also found that poor waste mismanagement (19) poses a threat to the physical and mental health of the slum inhabitants both immediately and in the future. According to the research, people who live in slums are more likely to contract a wide range of illnesses, such as the common cold, a headache, a skin disease, an infection of the urine, cancer, jaundice, pneumonia, and typhoid (7, 13, 22).

**Figure 1:**
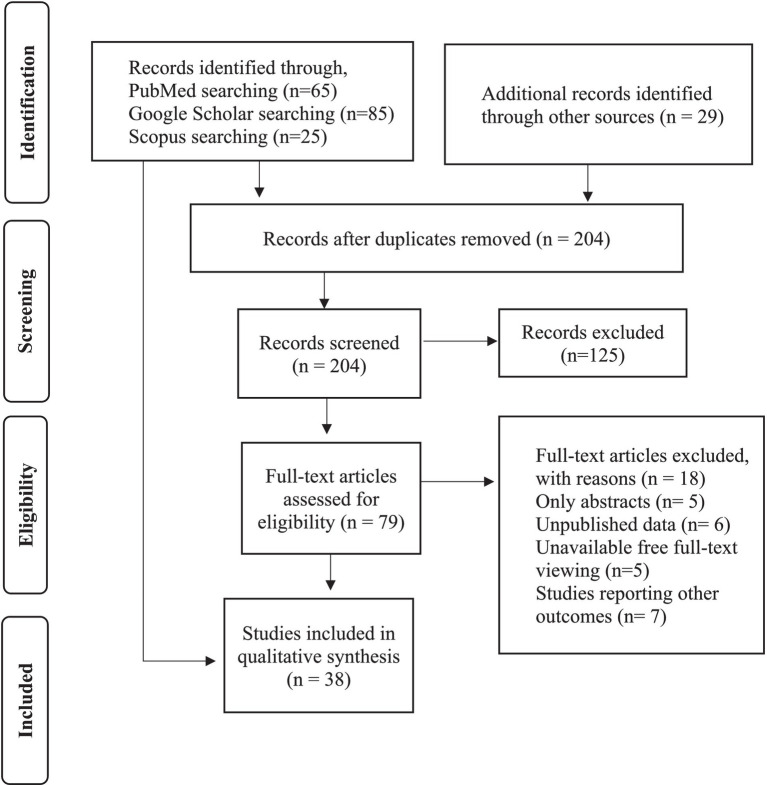
PRISMA flowchart describing the search strategy and inclusion/exclusion of studies for review (24).

As the world’s population is increasingly concentrated in cities, how to improve the quality of life of urban residents and protect their health is crucial (17, 23). Both communicable and non-communicable diseases (6) have increased due to poor urban planning and governance (13) because of the disparate access to health-promoting resources and the accumulation of risks that influence the health of different population groups. At the same time, it is understood that the urban environment is not a static, uniform whole, but rather a dynamic, interconnected web of various urban structures (e.g., those about education, economics, transportation, and politics). As a result, meaningful collaboration between many fields and sectors is necessary for urban health improvement and illness prevention (18) (see Figure 1).

To effectively address the health risks posed by unplanned urbanization in Dhaka, understanding the complex relationship between the urban environment and public health is crucial. Success will require excellent urban planning and governance, the sharing of best practice models, and the commitment and leadership of stakeholders across disciplines, sectors, communities, and countries (19, 25). This research paper aims to uncover the associations between unplanned urbanization and specific health outcomes in Dhaka City by conducting a comprehensive literature review and analysis of existing studies. By synthesizing the available evidence, this study seeks to provide insights into the causal pathways linking urban environment factors, such as air pollution, water and sanitation issues, and socioeconomic determinants, with public health outcomes in the context of unplanned urbanization. However, the unplanned urbanization of Dhaka City has resulted in significant health risks and challenges for its residents. Addressing these issues requires a holistic approach that incorporates effective urban planning, environmental management, and equitable access to healthcare services. By uncovering the associations between unplanned urbanization, the urban environment, and public health, this research paper aims to contribute to the growing body of knowledge on this critical topic and inform evidence-based interventions and policies to improve the health and well-being of the urban population in Dhaka City (see Table 1).

**Table 1:** Analyzing the critical reflection of influential scholarly research findings and gaps.

**#**	Study details	Critical reflection of research findings and gaps
1.	**Authors:** Ribeiro (1) **Title:** Unplanned Urban Development: A Neglected Global Threat **Methodology/Approaches:** Narrative Literature Review **Journal/Source:** *Current Urban Studies*	The research findings underscore the critical importance of considering urbanization and disaster resilience. They highlight that urban growth, when not adequately planned, significantly contributes to vulnerability in cities like Bangalore, India, and São Paulo, Brazil. This draws attention to the often-overlooked global threats cities face and emphasizes the need for comprehensive disaster mitigation strategies in urban development. However, there’s a notable research gap in understanding how these insights apply to rapidly urbanizing areas like Dhaka City, where unique challenges and vulnerabilities may exist.
2.	**Authors:** Alirol et al. (2) **Title:** Urbanisation and infectious diseases in a globalized world **Methodology/Approaches:** Review paper **Journal/Source:** *The Lancet infectious diseases*	Urbanization is a global phenomenon. The United Nations projects that by 2050, metropolitan areas will be home to 6.3 billion people, up from 3.3 billion in 2007. This urban growth, particularly pronounced in underdeveloped nations, poses significant challenges to global public health. Recent pandemics have underscored cities as hotspots for the spread of communicable diseases, attributed to heightened international travel and migration. While this study emphasizes the impact on public health, there is a research gap in examining how urbanization specifically influences disease spread in densely populated and rapidly urbanizing cities like Dhaka.
3.	**Authors:** Graham et al. (3) **Title:** Peri-urbanization and in-home environmental health risks: the side effects of planned and unplanned growth **Methodology/Approaches:** Quantitave; survey method **Journal/Source:** *International Journal of Hygiene and Environmental Health* (Elsevier)	A Northern Mexican metropolis serves as a case study to investigate environmental health hazards arising from urban development. Both planned and unplanned communities exhibit vulnerabilities to poor environmental health conditions, with unplanned areas showing stronger associations with risk factors and negative health outcomes. In Dhaka City, there’s a notable research gap in understanding how rapid urbanization and the stark differences between planned and unplanned areas impact environmental health hazards and their associated health outcomes.
4.	**Authors:** Kondo et al. (5) **Title:** Urban Green Space and Its Impact on Human Health **Methodology/Approaches:** Systematic review **Journal/Source:** *International journal of environmental research and public health*	The expected urban population surge by 2050 highlights the significance of urban green spaces for well-being. Positive links between green space exposure and health indicators like mood and physical activity are promising, alongside negative links with mortality, heart rate, and violence, suggesting potential public health advantages. While green spaces are associated with better health, varying effects in studies suggest factors like green space quality, accessibility, personal preferences, and socioeconomics play a role. Understanding the intricate relationships between urban green spaces and public health requires further investigation.
5.	**Authors:** Lai et al. (6) **Title:** The impact of green space and biodiversity on health **Methodology/Approaches:** Review paper **Journal/Source:** *Frontiers in Ecology and the Environment*	In a meta-analysis encompassing studies on green space and health or biodiversity, most reports indicated positive effects, but a significant minority (22%) found no or even negative impacts on health. Notably, while green spaces are often associated with increased biodiversity, there’s a surprising lack of research concurrently exploring the health benefits and biodiversity of green spaces. The mechanisms linking urban green spaces and health need further elucidation, particularly concerning the role of microorganisms in influencing the immune system.
6.	**Authors:** Li et al. (26) **Title:** Urbanization and health in China, thinking at the national, local, and individual levels **Methodology/Approaches:** Review paper **Journal/Source:** *Environmental Health*	The rapid urbanization and changing urban habits in China have significant impacts on public health. China’s transition from infectious to chronic diseases has been swift, with environmental conditions, notably air and water pollution, playing a major role in disease and mortality. Key factors affecting urban health in China include an aging population, the food assistance system, and disparities in access to public health services, particularly among migrant workers. Further research is needed to understand and address these dynamics from the context of Bangladesh.
7.	**Authors:** Nnebue et al. (8) **Title:** Urbanization and health: An overview **Methodology/Approaches:** Review **Journal/Source:** *Orient Journal of Medicine*	Urban health is shaped by a multitude of factors, from the urban environment to access to social services and public transport. Despite a global shortage of reliable urban health data, available information indicates that urban health risks cut across diverse domains like medicine, ecology, energy, transportation, and urban planning, emphasizing the multidimensional nature of urban health challenges. Although reliable urban health data is scarce globally, existing information suggests that urban health risks span multiple domains, underscoring the multifaceted nature of urban health challenges.
8.	**Authors:** Moore et al. (9) **Title:** Global urbanization and its impact on health **Methodology/Approaches:** Narrative review **Journal/Source:** *International journal of hygiene and environmental health*	Urbanization attracts nearly half of the world’s population with promises of higher incomes, employment opportunities, quality healthcare, and cultural richness. Yet, rapid and uncontrolled urban growth often leads to poverty, ecological decline, and unmet service needs, posing health risks. Unfortunately, accurate urban health data, particularly at the neighborhood level, remain scarce, limiting our understanding of urban health challenges.
9.	**Authors:** Vardoulakis et al. (10) **Title:** Challenges and opportunities for urban environmental health and sustainability: the HEALTHY-POLIS initiative **Methodology/Approaches:** Review **Journal/Source:** *Environmental Health*	Due to rapid urbanization and growing lifestyles, more and more countries are facing environmental threats. Due to rapid urbanization and growing lifestyles, more and more countries are facing environmental threats. The main objective of the consortium is to foster and facilitate international research collaborations between two or more countries. The institute has expertise in environmental epidemiology, modeling, exposure assessment, system dynamics, and health impact assessment.
10.	**Authors:** McMichael (11) **Title:** Climate change-related migration and infectious disease **Methodology/Approaches:** Narrative review **Journal/Source:** *Virulence*	Human-induced climate change is poised to significantly impact human migration and population health, particularly regarding infectious diseases. Migration patterns will change, leading to shifts in infectious disease dynamics. This article underscores the potential influence of climate-induced migration on the spread of infectious diseases, including forced displacement, slow-onset migration to urban-poor areas, planned relocations, and labor migration tied to climate adaptation measures, all posing disease risks.
11.	**Authors:** McMichael (13) **Title:** The urban environment and health in a world of increasing globalization: issues for developing countries **Methodology/Approaches:** Editorial **Journal/Source:** *Bulletin of the world Health Organization*	Historically, urban environments have facilitated the spread of infectious diseases. However, in industrialized nations, there has been a shift from early *laissez-faire* urban expansion to collective urban management. Factors such as environmental degradation, improved education, democratic governance, and economic prosperity have contributed to this shift. Yet, in many low-income nations, progress is hindered by the demands and objectives of economic globalization.
12.	**Authors:** Arfanuzzaman & Dahiya (14) **Title:** Sustainable urbanization in Southeast Asia and beyond Challenges of population growth, and use change and environmental health **Methodology/Approaches:** Review; using multidisciplinary methods and techniques **Journal/Source:** *Growth and Change*	This research aimed to promote collaborative urban development by identifying commonalities among the studied cities. The findings reveal that urbanization in Southeast Asia is marked by haphazard and unplanned growth, disregarding the natural environment and ecological systems. These trends raise concerns about the well-being of present and future generations in Southeast Asian cities, including Dhaka, highlighting the need for strategic urban planning and sustainability efforts.
13.	**Authors:** Saravanan et al. (15) **Title:** Urbanization and human health in urban India: Institutional analysis of water-borne diseases in Ahmedabad. **Methodology/Approaches:** Quantitative; household surveys **Journal/Source:** *Health policy and planning*	Certain diseases are rapidly spreading in cities, especially waterborne infections, which thrive in areas with crumbling infrastructure, inequality, weak urban governance, growing economies, and mobile, crowded populations. This article explores the role of institutions as intermediaries in the spread of waterborne diseases in Ahmedabad, India. The research underscores how social actors exploit systemic inefficiencies, posing significant threats to urban health.
14.	**Authors:** Pradhan et al. (16) **Title:** Urban Growth and Environment and Health Hazards in Kathmandu Valley, Nepal **Methodology/Approaches:** Quantitative; survey questionnaire-based data **Journal/Source:** *Urban health risk and resilience in Asian cities* (Springer)	The rapid urban growth at Kathmandu valley in Nepal presents significant environmental and public health challenges, which this study seeks to quantify. Air and water pollution, along with inadequate sanitation, are major contributors to illness and mortality in the valley’s cities. As urbanization progresses, newly developed areas have encroached upon agricultural, forested, public, and open spaces without proper planning.
15.	**Authors:** Adams et al. (17) **Title:** Who serves the urban poor? A geospatial and descriptive analysis of health services in slum settlements in Dhaka, Bangladesh **Methodology/Approaches:** Quantitative; descriptive statistics **Journal/Source:** *Health policy and planning*	In Bangladesh, urbanization’s health challenges disproportionately impact the urban poor. A significant portion of the private healthcare sector includes consultation rooms (20%) pharmacies and alternative practitioners (75%). Despite the need for continuous healthcare access, many clinics, including those backed by projects like the Urban Primary Health Care Project (UPHCP) and NGOs, offer limited hours, often in the late afternoon. Consequently, private Health Service Delivery Providers (HSDPs) provide after-hours care, despite only 37% of their staff having official medical degrees, raising concerns about care quality.
16.	**Authors:** Byomkesh et al. (18) **Title:** Urbanization and green space dynamics in Greater Dhaka, Bangladesh **Methodology/Approaches:** Quantitative; using spatial metrics **Journal/Source:** *Landscape and Ecological Engineering*	In urban environments, green spaces play a vital role in maintaining the local ecosystem’s health. This research aimed to create a dynamic map of green areas in Greater Dhaka and monitor their status. Despite the numerous ecological, economic, and social benefits these spaces offer, the study reveals a gradual decline in their presence over time. The growth of the urban population, driven primarily by rural-to-urban migration, is identified as a major factor in the reduction of green areas.
17.	**Authors:** Islam et al. (19) **Title:** Environmental perception during rapid population growth and urbanization: a case study of Dhaka city **Methodology/Approaches:** Quantitative; questionnaire-based factual and perceptual data **Journal/Source:** *Environment, development and sustainability*	Dhaka, Bangladesh’s capital, has witnessed a swift surge in population and urbanization. The city’s rapid growth, fueled by population increase, unplanned urban development, and peripheral industries, has significantly altered the land use pattern, resulting in extensive urban expansion. Unfortunately, the environmental consequences of this expansion are often overlooked as it encroaches upon farmland, forests, wetlands, and water bodies. This research argues that Dhaka city’s residents are not experiencing an equivalent level of environmental change or its associated challenges.
18.	**Authors:** Hasan et al. (20) **Title:** Prevalence and associated factors of hypertension in selected urban and rural areas of Dhaka, Bangladesh: findings from SHASTO baseline survey **Methodology/Approaches:** Quantitative; cross-sectional study **Journal/Source:** *BMJ open*	This research aimed to assess the prevalence of hypertension and its associated risk factors among adult males and females in urban and rural areas of Bangladesh’s Dhaka division. The study found that hypertension was prevalent in 31.0% of the population, with higher rates among urban residents (36.9% compared to 30.0% in rural areas). Older women were identified as having a higher risk of hypertension compared to younger men, regardless of location, with adjusted odds ratios (AORs) of 1.3 and 1.7, respectively. Unmarried women in rural areas (AOR = 1.8) and unemployed women in urban areas (AOR = 1.7) were also more likely to have hypertension compared to married women.
19.	**Authors:** Uddin et al. (21) **Title:** Changes in urbanization and urban heat island effect in Dhaka city **Methodology/Approaches:** Quantitative; using Moderate Resolution Imaging Spectroradiometer (MODIS) **Journal/Source:** *Theoretical and Applied Climatology*	This research aimed to investigate the impact of urban heat islands (UHIs) on Dhaka City, Bangladesh, spanning from 2001 to 2017. During this period, Dhaka City experienced a 75.65% increase in population, accompanied by a 25.33% expansion in land area. Consequently, due to urbanization and higher population density, some parts of Dhaka City now have average temperatures of approximately 3 degrees Celsius higher than the city’s outskirts. Over 17 years, the city has witnessed a rise in both daytime and nighttime temperatures, with an increase of 0.03°C during the day and 0.023°C during the night.
20.	**Authors:** Baumgart et al. (25) **Title:** Urban development and public health in Dhaka, Bangladesh **Methodology/Approaches:** Narrative review **Journal/Source:** *Health in megacities and urban areas*	The relationship between socio-spatial development and public health in rapidly growing cities is particularly evident in developing regions. Local governments in these areas often face the challenge of meeting the needs of a growing population and economy while mitigating negative health impacts. In response, the World Health Organization (WHO) launched the “Urban planning as a critical link to building a healthy 21^st^ century” campaign in 2010, highlighting the importance of urban planning in addressing these health challenges.
21.	**Authors:** Islam et al. (27) **Title:** Assessing the relationship between COVID-19, air quality, and meteorological variables: a case study of Dhaka City in Bangladesh **Methodology/Approaches:** Quantitative; **Journal/Source:** *Aerosol and Air Quality Research*	This study in the Dhaka metropolitan area of Bangladesh explored the relationship between daily COVID-19 cases and specific air pollutants and weather conditions. The findings revealed significant correlations, particularly with relative humidity (lag 0 days) and pressure (lag 14 days), as indicated by LRM data. The GAM model further highlighted a nonlinear association between daily COVID-19 cases and meteorological and air quality factors at different lag times.
22.	**Authors:** Imran et al. (28) **Title:** Urban development and public health in Dhaka, Bangladesh **Methodology/Approaches:** Quantitative; Sensing (RS) and Geographic Information System (GIS) techniques **Journal/Source:** *Earth Systems and Environment*	The transformation of land use due to infrastructure development in urban areas leads to significant increases in Land Surface Temperature (LST). This study found that LST increased by an average of 6.43 degrees Celsius, with an annual increase of approximately 0.24°C. This temperature rise is linked to both developed and undeveloped land, resulting in a shift from moderate to severe heat stress for humans.
23.	**Authors:** Nahar et al. (29) **Title:** Assessment of the environmental perceptions, attitudes, and awareness of city dwellers regarding sustainable urban environmental management: A case study of Dhaka **Methodology/Approaches:** Quantitative; questionnaire survey **Journal/Source:** *Environment, Development and Sustainability*	Environmental challenges in emerging country cities are significant hurdles to sustainable urban development. Survey results indicate that respondents had a fair understanding of the causes of environmental contamination and expressed intentions to reduce pollution. Notably, demographic variables like age, education, occupation, and income showed statistically significant score differences, except for gender. Surprisingly, only 18% of respondents were aware of trash collection policies in their homes or neighborhoods.
24.	**Authors:** Hossain & Huggins. (30) **Title:** The Environmental and Social Impacts of Unplanned and Rapid Industrialization in Suburban Areas: The Case of the Greater Dhaka Region, Bangladesh **Methodology/Approaches:** Qualitative; using case study and KII **Journal/Source:** *Environment and Urbanization ASIA* (SAGE)	This research investigates how unchecked industrialization and a lack of regulatory policies drive unplanned urbanization in suburban areas, leading to environmental, infrastructural, and cultural consequences. The natural ecosystem suffers as land is cleared for factories and housing. Despite urban expansion, basic services and amenities remain inadequate. The absence of a planning body and development guidelines exacerbates the issue.
25.	**Authors:** McQuade et al. (31) **Title:** Real-time sewage surveillance for SARS-CoV-2 in Dhaka, Bangladesh versus clinical COVID-19 surveillance: a longitudinal environmental surveillance study (December 2019–December 2021). **Methodology/Approaches:** Quantitative; **Journal/Source:** *The Lancet Microbe*	This study observed SARS-CoV-2 presence in all income-level wards, with varying reported cases and durations. Ward 19, a wealthy area with high testing rates, had the most cases (47,683), significantly above the national average. From July to December 2021, the correlation between sewage viral load and clinical cases increased from r = 0.59 to r = 0.90. A concerning trend emerged as sewage samples showed viral load spikes 1–2 weeks before clinical cases, raising fears of an impending epidemic.
26.	**Authors:** Khandker et al. (32) **Title:** Air Pollution in Bangladesh and Its Consequences **Methodology/Approaches:** Narrative review **Journal/Source:** *Pre-print copy (Research Square)*	This research reviewed the literature on air pollution’s impact on health in Bangladesh. Factors such as congestion, fuel contamination, two-stroke auto rickshaws, overloading, and road dust contribute to transportation-related pollution. Additionally, city factories and brick kilns on the outskirts worsen air quality. Dhaka’s air pollution has increased, resulting in adverse health effects and environmental degradation.
27.	**Authors:** van der Heijden et al. (33) **Title:** Working to stay healthy’, health-seeking behavior in Bangladesh’s urban slums: a qualitative study **Methodology/Approaches:** Qualitative; using IDI and KII **Journal/Source:** *BMC Public Health*	Data analysis revealed that in Kamrangirchar and Hazaribagh of Dhaka City, people highly value their health as a crucial resource for work and survival. Local drugstores are relied upon for common health issues, and individuals navigate a fragmented health system to find quick solutions that minimize work disruptions. Those with serious illnesses often encounter skepticism, lack of understanding, and unfavorable outcomes when seeking medical care.
28.	**Authors:** Shafique et al. (34) **Title:** Right to health and social justice in Bangladesh: ethical dilemmas and obligations of state and non-state actors to ensure health for urban poor **Methodology/Approaches:** Narrative Literature Review **Journal/Source:** *BMC Medical Ethics*	The study focused on the ethical challenges within Bangladesh’s healthcare system amid global urbanization. It emphasized the urban–rural gap and the lack of collaboration among implementing organizations. The unchecked growth of the private sector and unethical business practices contribute to debt accumulation and deteriorating living conditions among the urban poor.
29.	**Authors:** Rashid et al. (35) **Title:** Urban poverty, climate change, and health risks for slum dwellers in Bangladesh **Methodology/Approaches:** Quantitative; spatial mapping based study **Journal/Source:** *Climate change adaptation actions in Bangladesh* (Springer)	In the 21st century, the well-being and rights of urban slum dwellers are critically important due to the growing threats posed by climate change. While rural areas are more susceptible to climate change-related disasters, urban centers are not immune. In urban slums, families take measures like elevating their mattresses with bricks or moving to higher floors, when possible, driven by the need for stronger roofs. However, overcrowding and inadequate infrastructure make the situation particularly challenging for the urban poor.
30.	**Authors:** Alam et al. (36) **Title:** Unplanned urbanization: Assessment through calculation of environmental degradation index. **Methodology/Approaches:** Quantitative; survey data. **Journal/Source:** *International Journal of Environmental Science and Technology* (Springer)	Bangladesh, like many developing countries, is undergoing urbanization as people migrate from rural areas to cities due to a mix of push and pull factors. However, this rapid and unplanned urbanization is causing environmental degradation. Specific locations are experiencing higher-than-permitted concentrations of dust, SO2, and NO2. Additionally, the city of Sylhet in Bangladesh has witnessed a substantial decline in its groundwater level over the past few decades, reaching a new low of 7,880 mm below ground from 3,380 mm in 1982.
31.	**Authors:** Roy, S. and Sowgat, T. (4) **Title:** Sustainable Urbanisation in Bangladesh and Dhaka: Challenges and Way Forward **Methodology/Approaches:** Review **Journal/Source:** Background Paper 23rd ASEF Summer University ASEF Education Department October 2021	Bangladesh’s rapid urbanization poses significant challenges to Dhaka, its capital city. Between 1991 and 2019, Dhaka experienced an average annual urban growth rate of 8%, with its outskirts expanding at an alarming rate of 43% during the same period. Specifically, the built-up areas in Dhaka’s outskirts expanded by 234 km2, surpassing the city’s growth of approximately 116 km2 at the same time. The growth has strained urban services and created socio-spatial divisions. To address these issues, Dhaka needs policies that prioritize decentralization and sustainability over growth.
32.	**Authors:** Roy, S., Basak, D., Bose, A., et al. (37) **Title:** Citizens’ perception toward landfill exposure and its associated health effects: a PLS-SEM based modeling approach. **Methodology/Approaches:** PLS-SEM-based modeling approach **Journal/Source:** *Environmental Monitoring and Assessment*, (PubMed)	Improper treatment of approximately 90% of municipal solid waste in India, often disposed of in open dumps and landfills, presents a grave threat to public health. These landfills are a constant source of inconvenience, leading to feelings of uncertainty, stress, and dissatisfaction among residents in nearby areas. The research findings underscore the significant impact of three key factors: environmental quality (β = 0.997, t = 19.607, and ρ < 0.001), overall life satisfaction (β = 0.116, t = 2.475, and ρ < 0.05), and individuals’ defensive attitudes (β = 0.150, t = 2.526, and ρ < 0.001) on the health of those exposed to landfill sites.
33.	**Authors:** Roy, S., et al. (38) **Title:** Evaluating urban environment quality (UEQ) for Class-I Indian city: an integrated RS-GIS-based exploratory spatial analysis **Methodology/Approaches:** exploratory Spatial Data Analysis, multivariate model **Journal/Source:** *GEOCARTO INTERNATIONAL*, (Taylor & Francis)	The spatial distribution of urban environmental quality and the constructed domains (except socio-economy) demonstrated that areas with Low-Low values were primarily clustered in the city center. In contrast, High-High patterns were predominantly concentrated in the outskirts. Additionally, the calculation of Moran’s I value indicated the presence of spatial autocorrelation and a non-random pattern within Siliguri City. These findings from the analysis reveal significant spatial variations and distinctions across the study area.
34.	**Authors:** Roy, S. et al. (23) **Title:** Does Geographical Heterogeneity Influence Urban Quality of Life? A Case of a Densely Populated Indian City **Methodology/Approaches:** Exploratory spatial data analysis **Journal/Source:** *Papers in Applied Geography* (Taylor & Francis)	The rapid growth of urban populations and urbanization in Indian cities has posed significant challenges to maintaining the urban quality of life (QoL). Spatial heterogeneity, which refers to the varying quality of life experienced by people in different geographic units within a city, such as neighborhoods or wards, has become a notable concern. The research findings underscore the alarming overall poor quality of life in Siliguri, with evident geographic disparities.
35.	**Authors:** Majumder, **S.** et al. (39) **Title:** Multiscale GIS based-model to assess urban social vulnerability and associated risk: Evidence from 146 urban centers of Eastern India **Methodology/Approaches:** The multiscale geographically weighted regression (MGWR) **Journal/Source:** *Sustainable Cities and Society* (Elsevier)	This study focuses on assessing social vulnerability in urban areas, emphasizing the various forms of social deprivation experienced by city residents. Urban social vulnerability (USoV) is broken down into three interconnected dimensions: exposure, sensitivity, and adaptive capacity. The results reveal that a majority of urban areas face moderate to high levels of exposure and sensitivity, coupled with low adaptive capacity, indicating a high level of USoV. Furthermore, the analysis shows that adaptive capacity demonstrates the strongest spatial association, while sensitivity exhibits the lowest Moran value and, consequently, the weakest association.
36.	**Authors:** Roy, S. et al. (12) **Title:** Urban waterlogging risk as an undervalued environmental challenge: An Integrated MCDA-GIS based modeling approach **Methodology/Approaches:** Systematic Waterlogging Assessment **Journal/Source:** *Environmental Challenges* (Elsevier)	The results reveal that a significant portion of Siliguri, approximately 46%, falls within high to very high waterlogging hazard zones, with 38% being highly vulnerable to waterlogging. Moreover, the risk map highlights that around 35% of the city faces a threat of waterlogging, primarily concentrated in the central city area. The study’s model demonstrated good accuracy, with an area under the curve (AUC) of 78.2%. Overall, this research provides a valuable strategy that can inform planning and mitigation efforts to reduce waterlogging incidents not only in Siliguri but also in similar urban areas globally like Dhaka City.
37.	**Authors:** Roy, S., and Singha, N. (40) **Title:** Reduction in concentration of PM2.5 in India’s top most polluted cities: with special reference to post-lockdown period **Methodology/Approaches:** Quantitative Approach and Trend Analysis **Journal/Source:** *Air Quality, Atmosphere & Health* (Springer)	The study analyzes the impact of India’s COVID-19 lockdown, which lasted over two months and curbed mass activities contributing to air pollution. It focuses on PM2.5 levels in the top ten polluted Indian cities, dividing the 7 months into pre-lockdown, lockdown, and post-lockdown phases. Findings indicate that PM2.5 concentrations dropped significantly during the lockdown, with post-lockdown levels remaining below national standards.
38.	**Authors:** Roy, S. and Mandal, G. (22) **Title:** The Problems of Older Adult Residing in Slums: A Case Study on Siliguri Municipality **Methodology/Approaches:** Quantitative **Journal/Source:** Indian Journal of Gerontology	This study examined the issues faced by 200 older adult individuals (114 males, 86 females) aged 60 and above in 10 Siliguri slums. Health problems affected 40%, financial troubles 24.5%, social difficulties 15.5, and 1.5% faced other issues. Gender was significantly associated with problem types. Recommendations include state and societal measures to address the needs of these older adult residents.

## 2. Research design and methodology

### 2.1. Research design

This research paper adopts a comprehensive systematic literature review and analysis approach which is a structured and comprehensive process aimed at identifying, collecting, evaluating, and synthesizing existing research on a specific topic or research question. It involves a series of structured steps that include formulating research questions or objectives, creating a systematic search strategy, searching relevant literature sources, screening and selecting studies based on predefined criteria, extracting essential data from the chosen studies, assessing study quality, synthesizing findings, and evaluating the potential for publication bias. The objective of this review to uncover the associations between unplanned urbanization, the urban environment, and public health in Dhaka City, Bangladesh. The research questions guiding this review are as follows: (a) What are the key health risks associated with unplanned urbanization in Dhaka City? (b) What are the specific urban environment factors contributing to these health risks? (c) How do socioeconomic determinants influence the relationship between unplanned urbanization and public health outcomes in Dhaka City?

### 2.2. Methodology

#### 2.2.1. Search strategies

This study adhered to PRISMA guidelines (24), employing a systematic approach that encompassed defining research questions and inclusion/exclusion criteria, evaluating selected studies on ‘Urban Environment and Public Health,’ extracting pertinent data, assessing quality and bias risk, synthesizing findings, and providing clear and structured results, interpretations, discussions, implications, and limitations. The study followed a well-structured process, as outlined in PRISMA guidelines, to ensure a comprehensive and rigorous review of the literature on this topic. Moreover, a review research design involves synthesizing and analyzing existing literature, reports, and studies to provide an overview of the topic and identify key findings and gaps in knowledge. Databases such as PubMed, Scopus, Springer, Elsevier, BMJ and Google Scholar are searched using keywords such as “unplanned urbanization,” “urban environment,” “public health risks,” “Dhaka City,” “Bangladesh,” and related terms. Additional sources, such as government reports, policy documents, and grey literature, are also consulted to ensure a comprehensive review.

#### 2.2.2. Inclusion and exclusion criteria

**Figure 2:**
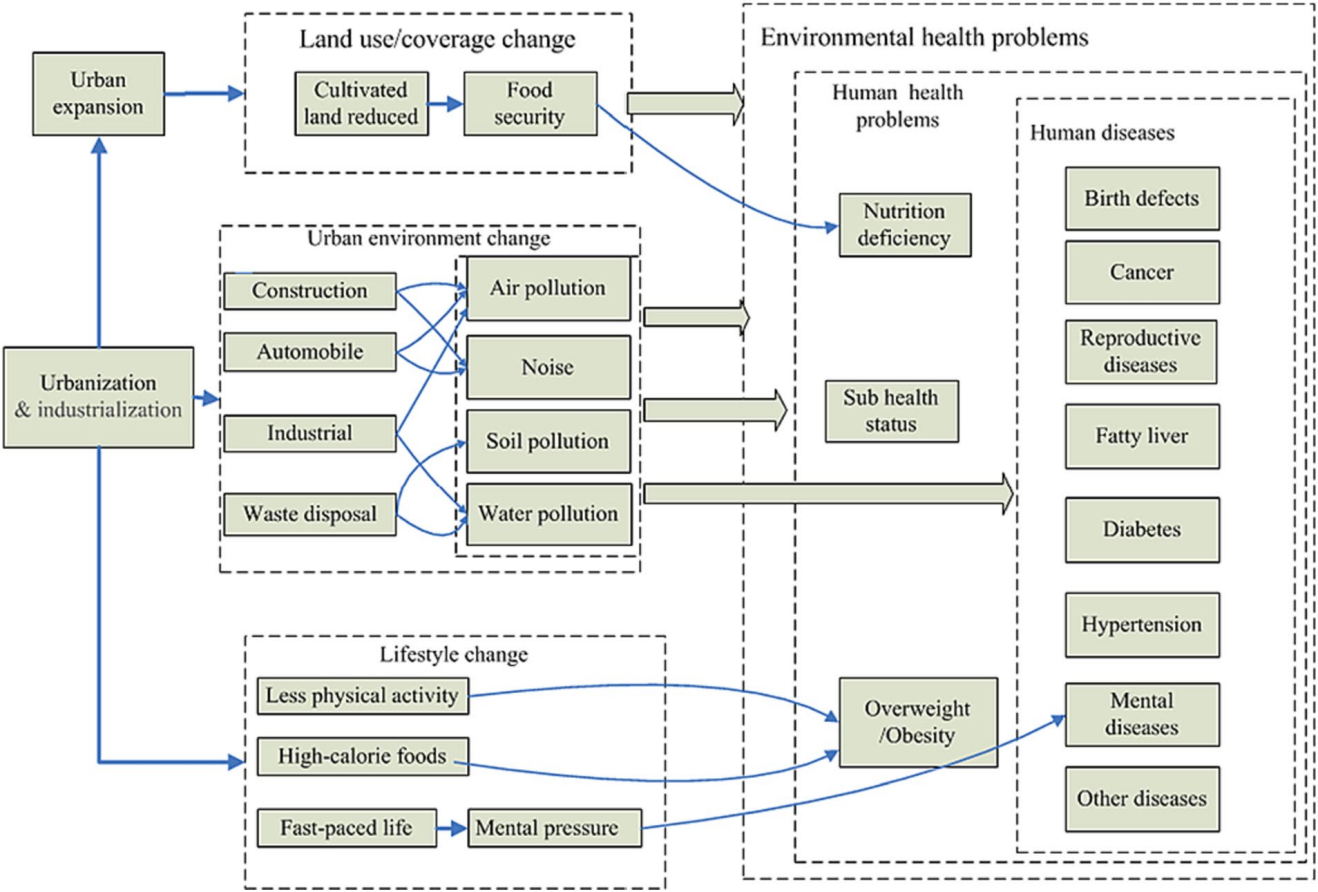
Associations between Urban Environment and Public Health [Adapted from (26)].

Inclusion measures intended for choosing lessons include those that focus on the health risks associated with unplanned urbanization in Dhaka City, as well as studies that examine the relationships between the urban environment and public health outcomes. Finally, we included 38 scholarly research papers (and additional six papers for other purposes) for this study collected from Google Scholar, Springer, Elsevier, Scopus, PubMed, BMJ etc. To ensure relevance, studies for this review are selected based on criteria including study design, geographic focus (Dhaka City), publication date, and language. This process helps identify and include studies closely aligned with the research question while excluding those conducted in other locations or beyond the specified timeframe. Besides, the PRISMA model uses exclusion criteria to filter studies based on factors like relevance, quality, language, publication date, geographic focus, study type, methodology, sample size, data availability, duplication, accessibility, and more (24). These criteria help ensure that selected studies align with the research objectives while excluding irrelevant or unsuitable ones (see Figure 2).

#### 2.2.3. Data extraction and analysis

**Figure 3:**
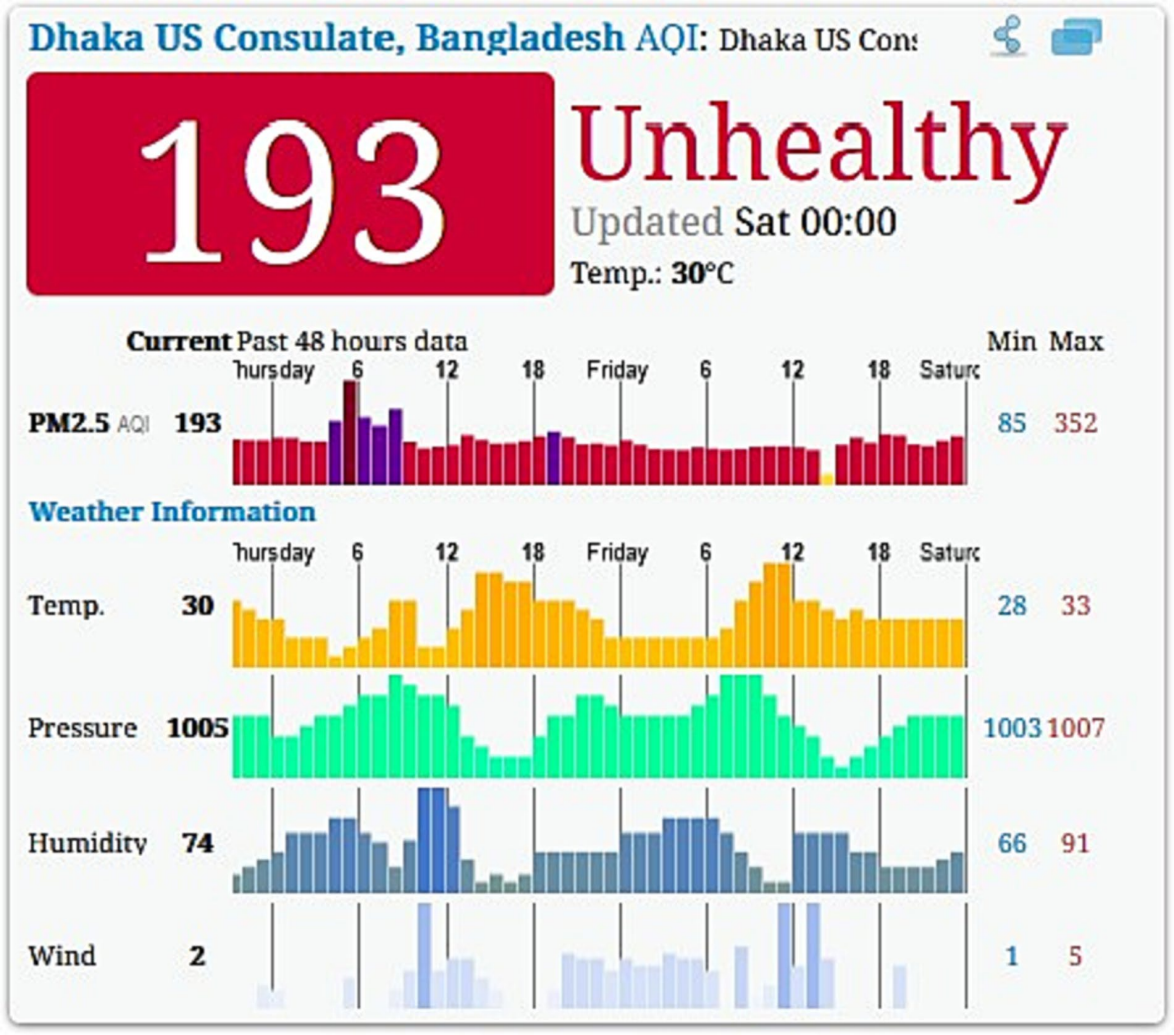
Dhaka US Consulate Air Pollution: Real-time Air Quality Index (AQI) (41). (Estimated on Saturday, September 2, 2023, 00.00).

In this study, a comprehensive search of academic databases was conducted to locate articles, reports, and studies related to the research question. The collected information included study objectives, the country of focus, population details, sample size, data sources, study periods, and health indicators used in the thematic analysis. Thematic analysis, a qualitative method used in this study, involves identifying and analyzing patterns or themes within textual or visual data. This process includes familiarizing with the data, initial coding, generating themes, refining them, defining, mapping, interpreting, and documenting these themes. It also involves extracting data, providing illustrative examples, cross-checking and comparing, writing up findings, and reflexivity in an iterative manner. The selected studies are thoroughly analyzed, and key information is extracted from them. This includes details such as the author(s), publication year, study objectives, study design, sample size, data collection methods, main findings, and the implications regarding the connections between unplanned urbanization and public health outcomes (see Figure 3).

#### 2.2.4. Quality assessment, synthesis, and interpretation

**Figure 4:**
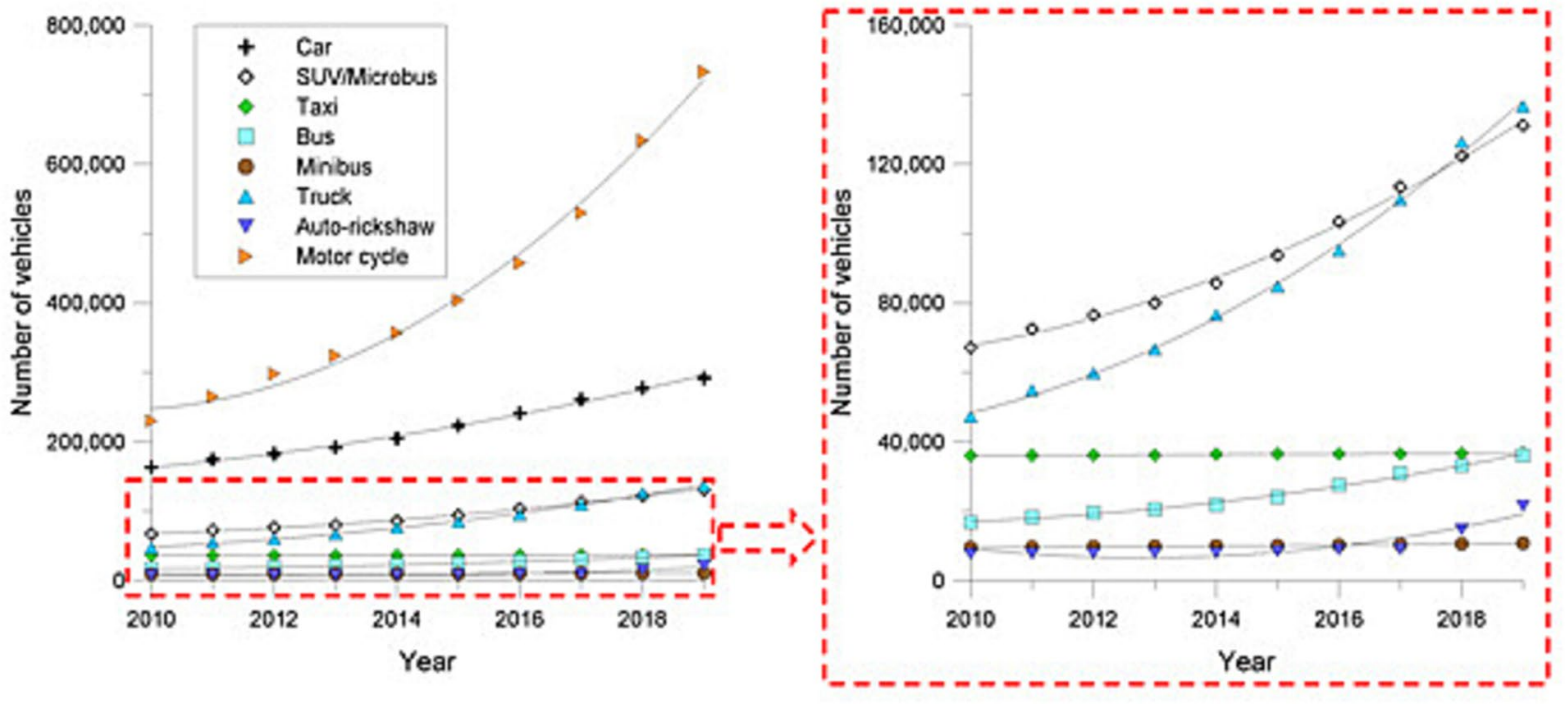
Indicating a growth pattern of the vehicles, the year-wise registered vehicles in Dhaka from 2010 to 2019 (42).

The quality and rigor of the studies included in the review are evaluated using established tools and criteria for reviewing research papers, such as the Preferred Reporting Items for Systematic Reviews and Meta-Analyses (PRISMA) guidelines (24). This assessment is essential to ensure that only high-quality research is considered, thereby enhancing the reliability and validity of the study results (see Figure 4).

### 2.3. Ethical considerations, strengths, and limitations

This study does not require ethical approval as it involves a systematic evaluation and analysis of existing research. Proper credit and citations will be given to all authors and sources. The paper’s strength lies in its systematic approach to examining the literature on the connections between unplanned urbanization, the built environment, and public health outcomes in Dhaka City. However, it is important to acknowledge the limitations of the methodology, including potential issues with sample bias, measurement accuracy, and generalizability in the underlying studies. Additionally, publication bias may exist due to accessibility and publishing factors. Nevertheless, this approach helps identify research gaps in Urban Public Health.

## 3. Discussion

### 3.1. Unplanned urbanization and health challenges faced by the population of Dhaka City

The results of this systematic review add to the ongoing conversation about the risks to public health posed by rapid or unplanned urbanization and the subsequent proliferation of Aedes-borne illnesses in densely populated areas (19). Disease dynamics in urban settings pose a serious challenge to public health systems and necessitate multifaceted, interdisciplinary methods for prevention and control. The combination of rapid, unplanned urbanization and other socioecological issues, such as climate and environmental change, has made many parts of the world more susceptible to the spread of vectors and illnesses (20). Dhaka City’s unplanned urbanization has resulted in an extensive assortment of health problems for its residents. The city’s rapid and unchecked expansion has led to several interconnected problems that threaten the lives of its citizens. Furthermore, informal settlements and slums have proliferated in Dhaka City due to uncontrolled urbanization. Conditions in these locations are characterized by high population density, a lack of acceptable housing options, and a scarcity of basic services. Infectious diseases, such as those spread through the air, water, or insects, are more likely to spread in overcrowded environments (22, 27).

However, the unplanned growth of the city has strained the existing sanitation infrastructure. Many areas lack proper sewage systems and waste management practices, leading to contamination of water sources and the spread of diseases like diarrhea, cholera, and hepatitis. The lack of proper waste disposal also contributes to environmental pollution and health hazards (12, 20). Moreover, the rapid population growth and unplanned urbanization have strained the healthcare infrastructure in Dhaka City. Many areas lack sufficient healthcare facilities, leading to inadequate access to primary healthcare services. This results in delayed or insufficient treatment of illnesses and limited preventive care, exacerbating the health challenges faced by the population (17). Accordingly, the majority of hospitals are privately owned, but the number of public and private units is roughly equal. This has led to an unequal distribution of health facilities and accommodations in urban and peri-urban regions (21).

Besides, unplanned urbanization has exacerbated socioeconomic disparities in Dhaka City. The unequal distribution of resources, limited job opportunities, and unequal access to education and healthcare services have created pockets of poverty and marginalization. Individuals from lower socioeconomic backgrounds face additional challenges in accessing healthcare, leading to poorer health outcomes (6, 28). Additionally, unplanned urbanization can contribute to mental health issues among the population of Dhaka City. The rapid pace of urbanization, social dislocation, and stressors associated with living in crowded and chaotic environments can increase the risk of mental health disorders such as anxiety and depression (25). Addressing these health challenges requires a comprehensive approach that integrates urban planning, infrastructure development, healthcare provision, and social interventions. It is essential to implement effective urban planning strategies that prioritize equitable access to housing, sanitation, and healthcare services. Improving waste management systems, promoting clean energy initiatives, and reducing vehicular emissions can help mitigate air pollution (20).

### 3.2. Urban environment factors contributing to public health risks

The population of Dhaka City in Bangladesh faces a range of health challenges due to various factors associated with urbanization. The high population density in Dhaka City, along with poor water and sewage systems, makes it more difficult to control the spread of disease. Diseases spread by water, such as cholera and typhoid, and by insects, such as dengue and malaria, represent serious threats to public health (27). Additionally, car emissions, industrial operations, and open rubbish burning all contribute to Dhaka City’s extremely unhealthy air quality. Asthma, bronchitis, and lung cancer are only some of the respiratory illnesses that are made worse by bad air quality (16). In addition, Dhaka City is known for its high noise levels due to heavy traffic, construction activities, and commercial areas. Prolonged exposure to excessive noise can lead to hearing loss, sleep disturbances, stress-related disorders, and cardiovascular problems (19). On the other hand, rapid population growth and migration have led to overcrowding in many areas of Dhaka City. This overcrowding is particularly prevalent in urban slums and informal settlements, where residents face poor living conditions, limited access to basic services, and increased health risks, including infectious diseases and inadequate healthcare (13).

Moreover, the rapid growth of Dhaka City has outpaced the development of healthcare infrastructure, resulting in a strain on the healthcare system. Hospitals and healthcare facilities often face overcrowding, insufficient resources, and limited accessibility, leading to challenges in providing adequate healthcare services to the population (7, 29). Consequently, Dhaka City faces the dual challenge of both undernutrition and overnutrition. Some populations suffer from malnutrition and food insecurity, while others face a rising prevalence of obesity and non-communicable diseases such as diabetes due to changing dietary patterns and sedentary lifestyles (16). In addition, the fast-paced urban environment, social pressures, and economic disparities in Dhaka City contribute to mental health issues. High levels of stress, anxiety, and depression are prevalent among the population, necessitating better access to mental health services and support (28).

Therefore, there are serious threats to people’s health in Dhaka City due to a lack of access to clean water and inadequate sanitary services. The prevalence of diarrheal disorders and other water-related infections (7) can be directly attributed to the prevalence of contaminated water sources and poor sanitation practices. Clean and safe drinking water is a problem in Dhaka City. Cholera, typhoid, and diarrhea are only some of the waterborne diseases that can spread due to contaminated water sources, inadequate water treatment facilities, and poor sanitation practices (13). At the same time, Dhaka city is affected by the urban heat island effect, and the large urban population density and abundance of man-made surfaces (such as buildings and concrete) cause the city’s temperature to rise above that of the surrounding countryside. There is an increase in the incidence of heat-related diseases and heat stress during the hot summer months (26, 27). Addressing these urban environment factors and mitigating the associated public health risks requires a comprehensive approach. It involves implementing effective urban planning strategies, improving air quality through emission controls and promoting green spaces, enhancing water and sanitation infrastructure, upgrading informal settlements, and providing equitable access to healthcare services. Additionally, public awareness campaigns and community participation are vital to promote healthy behaviors and create a sustainable and livable urban environment.

### 3.3. Social and environmental determinants of health in Dhaka City

The city of Dhaka, the capital of Bangladesh, is grappling with numerous social and environmental health determinants that seriously affect the well-being of its residents. As cities grow, factors such as the social environment, the natural environment, and the provision and access to health and social services affect the health of urban populations (25). Understanding and addressing these determinants are crucial for improving the overall health outcomes and quality of life in the city. Dhaka City faces numerous social and environmental factors that contribute to health risks among its population. Rapid urbanization, inadequate urban planning, and limited access to basic services create unfavorable living conditions. These factors include air pollution, water and sanitation issues, overcrowding, inadequate housing, limited access to healthcare, and insufficient green spaces. The interplay of these factors exacerbates health risks and negatively impacts the well-being of the population (18, 23).

High levels of particulate matter (PM), nitrogen dioxide (NO_2_), and sulfur dioxide (SO_2_) in the air have detrimental effects on respiratory health, and as a result, Dhaka City is plagued by severe air pollution caused primarily by industrial emissions, vehicular traffic, and the burning of solid waste. Asthma and chronic obstructive pulmonary disease (COPD) are just two of the many respiratory disorders linked to air pollution, according to research (16, 40). The health hazards associated with poor air quality can be greatly reduced if actions are taken to address air pollution. These actions include imposing rigorous controls on emissions, enhancing waste management, and promoting sustainable mobility (20). The inadequate water and sanitation infrastructure in Dhaka City poses serious threats to public health. Waterborne infections, such as diarrheal disorders, can spread quickly due to contaminated water supplies and a lack of proper sanitation infrastructure. Likewise, the spread of vector-borne diseases and the degradation of the natural environment are both exacerbated by insufficient attention to cleanliness and the management of waste (7, 13, 18). Providing clean water to drink, enhancing sanitation services, and establishing effective waste management systems are all important steps toward reducing these threats to public health (18).

Moreover, Dhaka City faces the challenges of rapid population growth and overcrowding, leading to substandard housing conditions for many residents. Overcrowded living spaces, lack of ventilation, and inadequate access to basic amenities contribute to the spread of infectious diseases and poor overall health outcomes (29). The city of Dhaka has been struggling with air pollution for years. Its air quality declines in the winter and improves during the monsoon. Dhaka had the third worst air quality of any major city in the world at 8:55 a.m., when the air quality index (AQI) recorded 193 (41). Dhaka City experiences substantial rural-to-urban migration (30) and rapid population growth, leading to overcrowding and strained infrastructure. The influx of migrants often settles in informal settlements and slums, lacking access to basic services and adequate living conditions (7). Addressing housing conditions through urban planning, affordable housing initiatives and slum redevelopment programs can improve living conditions and mitigate the health risks associated with overcrowding. Dhaka City exhibits significant socioeconomic disparities, with high levels of poverty and income inequality (30). These disparities play a crucial role in shaping health outcomes. Marginalized populations, such as the urban poor, face barriers to accessing quality healthcare, proper nutrition, and safe living conditions. Limited access to healthcare services, inadequate nutrition, and substandard living conditions contribute to adverse health outcomes (29). Addressing socioeconomic disparities through poverty alleviation programs, equitable access to healthcare, and social support systems can help reduce health inequities and improve overall health outcomes.

However, the scarcity of urban green spaces in Dhaka City is another important environmental determinant of health. In addition, limited access to parks, gardens, and recreational areas deprives residents of opportunities for physical activity, relaxation, and social interaction. Increasing the availability and accessibility of green spaces can enhance physical and mental health, reduce stress levels, and improve overall health. Consequently, land-use patterns in Dhaka City significantly influence the health of its residents. Unplanned urbanization and haphazard development lead to the encroachment of water bodies, reduction of green spaces, and increased pollution levels. The conversion of agricultural land to urban settlements disrupts the ecological balance and contributes to environmental degradation (9, 31, 37). However, these land-use patterns directly impact air quality, water availability, and the overall livability of the city, thereby affecting the health of the population. Inadequate urban infrastructure exacerbates health risks in Dhaka City. Limited access to clean water, sanitation facilities, and proper waste management systems contributes to the spread of waterborne diseases and environmental pollution (26, 32). Insufficient healthcare facilities and transportation infrastructure pose barriers to healthcare access. The absence of well-planned urban infrastructure further contributes to the burden of disease and hinders efforts to promote public health.

### 3.4. Resilience of urban slums and the risks of infectious diseases

The resilience of urban slums, while admirable in the face of adversity, can paradoxically increase the risk of infectious diseases for their inhabitants. Resilience is the ability of communities or populations to withstand and recover from stress that often manifests as the ability to adapt to challenging living conditions, and social and economic difficulties with limited resources (33, 34). However, slum populations can be transient, with frequent migration and mobility patterns facilitating the spread of infectious diseases between different communities and areas, making containment efforts more challenging (35). In Dhaka city, at least 4 million people living in more than 5,000 slums in Dhaka Metropolis are at long-term physical and mental health risk due to living in filthy environments. And it has increased domestic violence and social intolerance. Besides, there are social barriers such as slum children’s reluctance to go to school, and teenagers’ reluctance to go to education or work. Also, financial losses such as loss of income and increased medical expenses (36). Therefore, the resilience of Dhaka city’s urban slums and the vulnerability they face to communicable diseases is a serious issue that deserves attention. As the city experiences rapid and unplanned urbanization, the growth of informal settlements and slums has become a significant challenge (22, 30). Besides, these urban slums are characterized by overcrowded living conditions, inadequate sanitation facilities, and limited access to healthcare services, making their inhabitants highly vulnerable (36). One of the key factors contributing to the vulnerability of urban slum dwellers is the lack of proper housing and sanitation. Additionally, many slum dwellers live in makeshift shelters with limited access to clean water and sanitation facilities, creating an environment conducive to the threats to public health. Waterborne illnesses such as cholera, typhoid, and diarrheal diseases are prevalent in these areas due to contaminated water sources and poor sanitation practices (22, 31, 39).

In addition, the lack of proper ventilation and adequate waste management systems contributes to the transmission of airborne diseases such as tuberculosis. However, improper disposal of waste and lack of waste collection services can lead to environmental pollution, attracting disease vectors and promoting the spread of vector-borne infections (26, 32). Moreover, access to healthcare services is another critical aspect affecting the resilience of urban slum populations. Therefore, many slum dwellers face barriers in accessing healthcare facilities, including affordability, distance, and cultural barriers (28, 43). As a result, preventive and curative healthcare measures are often inadequate, leading to delayed diagnosis and treatment of severe diseases. Health literacy among slum dwellers may be low, leading to a lack of knowledge about preventive measures and early recognition of symptoms. Moreover, the transient nature of slum populations, with frequent migration and informal settlements, can hinder the effectiveness of public health interventions (30).

### 3.5. The uniqueness of Dhaka City’s challenges and implications of the findings

Dhaka City presents a unique set of challenges due to its rapid urbanization and population density. While drawing parallels with other rapidly urbanizing areas, it’s important to recognize Dhaka’s distinctiveness. The city faces extreme levels of air pollution, exacerbated by a high density of vehicles and industrial emissions. Its unplanned growth has led to overcrowding, inadequate infrastructure, and insufficient access to clean water and sanitation. The rapid growth of urban population and urbanization in Indian cities have created significant challenges in maintaining the quality of life (QoL) in urban and slum areas of Siliguri in particular (1, 13, 22, 23). Dhaka’s socioeconomic diversity further complicates public health interventions. Comparatively, other rapidly urbanizing areas may share some challenges, but Dhaka’s extreme concentration of issues demands tailored solutions that consider its specific context and complexities. The implications of the findings from Dhaka city’s study extend both within and beyond its borders. Within Dhaka, the findings shed light on the urgent need for comprehensive urban planning and public health interventions. High levels of pollution, inadequate sanitation, and socioeconomic disparities require targeted policies to improve living conditions (7, 12, 22, 23, 30, 32). Moreover, governance structures and policies need reforms to address the unique challenges of rapid urbanization. Beyond Dhaka, these findings serve as a case study for other rapidly urbanizing regions globally. Lessons learned in Dhaka can inform strategies to tackle similar issues in other cities grappling with unplanned urbanization and its associated health risks, providing valuable insights for policymakers and urban planners worldwide.

## 4. Policy and governance: implications and interventions

### 4.1. Healthier and sustainable urban environment

Unplanned urbanization in Dhaka City has led to significant health risks associated with the urban environment. Addressing these risks requires effective policy interventions and governance measures to create a healthier and more sustainable urban environment. Accordingly, implementing comprehensive urban planning strategies and land use regulations is crucial to manage urban growth and ensure sustainable development. This includes zoning regulations, building codes, and land use policies that promote public health and safety. Prioritizing green spaces, preserving natural habitats, and encouraging mixed land use can enhance the urban environment and reduce health risks (32). Moreover, investing in adequate infrastructure development is essential to address the health risks associated with unplanned urbanization. This includes improving transportation systems, ensuring access to clean water and sanitation facilities, and expanding healthcare infrastructure. Enhancing urban infrastructure will promote better public health outcomes and reduce the burden of communicable diseases.

Implementing strict regulations and policies to control air pollution is crucial to protect public health. This can involve measures such as reducing industrial emissions, promoting cleaner energy sources, improving vehicle emissions standards, and enhancing public transportation systems. Additionally, increasing green space and implementing urban greening initiatives can help reduce air pollution and improve air quality in Dhaka city. However, ensuring equitable access to healthcare services is essential to address health disparities in Dhaka City (13, 33). This can be achieved through the establishment of community clinics, mobile health units, and healthcare facilities in underserved areas.

Implementing slum upgrading programs and improving housing conditions in informal settlements are crucial interventions to reduce health risks. This can involve providing secure tenure, upgrading housing infrastructure, and improving access to basic services such as water, sanitation, and electricity. Dhaka city’s current transport scenario is extremely susceptible from an ecological standpoint. Based on measurements of Dhaka City’s transport sustainability, it has been determined that the transport footprint is seventy times greater than the bio-capacity (42). This is due only to the city’s extensive physical transportation network and the CO2 emissions from its automobiles. Constant delays and heavy traffic, poor public transport, unsafe walking conditions, and rising pollution levels are all symptoms of the current transport crisis (34, 42). Effective governance and intersectoral collaboration among government agencies, urban planners, healthcare providers, and community stakeholders are critical for addressing the health risks of unplanned urbanization. Coordinated efforts, policy coherence, and collaboration across sectors can help integrate health considerations into urban planning, infrastructure development, and public health interventions. On the other hand, by implementing these policy implications and interventions, Dhaka City can effectively address the health risks associated with unplanned urbanization. Creating a sustainable, equitable, and healthy urban environment requires a multi-faceted approach, combining policy interventions, infrastructure development, healthcare access, public awareness, and effective governance (30, 31). The health and safety of the Dhaka City populace will improve thanks to these initiatives. Vulnerabilities and anthropogenic risks (29), in turn, are caused by social, political, and economic pressures. Socially constructed vulnerabilities, such as unplanned urban development, have received far more attention from the disaster study community than the mechanisms underlying the generation of hazards (12, 16). A high population density, poor sanitary facilities, high levels of outdoor and indoor air pollution, proximity to the coast, and poor air quality all contribute to an already precarious situation. This should help ensure that efforts made in developing-world urban areas contribute to a preventative environmental health agenda that improves people’s health and well-being (30, 33).

### 4.2. Green spaces and their impact on mental and physical health

Green spaces, such as parks, gardens, and open natural areas, have a positive impact on both mental and physical health in Dhaka City. Green spaces provide opportunities for physical activities such as walking, jogging, cycling, and recreational sports (13). These activities promote physical fitness, help in weight management, and reduce the risk of chronic diseases like obesity, diabetes, and cardiovascular ailments. Accessible green spaces encourage people to engage in regular exercise, leading to improved overall physical health. Stress reduction and relaxation from the bustling urban environment, allow individuals to connect with nature and experience a sense of calmness and relaxation. Spending time in green environments has been shown to reduce stress levels, lower blood pressure, and decrease the production of stress hormones. This, in turn, has a positive impact on mental health by alleviating symptoms of anxiety, and depression, and improving overall well-being. Improved air quality plays a crucial role in mitigating the issue of pollution. Trees and plants in green areas help purify the air by absorbing pollutants and releasing oxygen. By improving air quality, green spaces contribute to better respiratory health and reduce the risk of respiratory diseases among residents (31).

Moreover, exposure to green spaces has been linked to improved mental health outcomes. Studies have shown that spending time in nature and green environments can reduce symptoms of depression, improve mood, boost self-esteem, and increase feelings of happiness and well-being. Green spaces provide a visually pleasing and calming environment that promotes relaxation, stress reduction, and rejuvenation. Green spaces are gathering places for communities, promoting social interaction and community involvement (35, 37). Parks and gardens provide opportunities for people to come together, engage in recreational activities, and build social connections. This sense of community and social support contributes to positive mental health outcomes and a sense of belonging. Cognitive benefits and attention restoration: Research indicates that exposure to nature and green spaces can enhance cognitive function and attention restoration. Spending time in green environments has been associated with improved concentration, increased creativity, and better academic performance (30). This is particularly relevant for students and individuals who need to focus on demanding cognitive tasks. Biodiversity and ecological balance support by providing habitats for various plant and animal species. Biodiversity conservation is essential for maintaining ecological balance and ecosystem services (20, 34). By preserving green spaces and protecting biodiversity, Dhaka City can ensure a sustainable environment that benefits both human health and the overall ecosystem. To maximize the impact of green spaces on mental and physical health in Dhaka City, it is crucial to prioritize the creation and maintenance of green areas, especially in densely populated and underserved neighborhoods. This includes establishing more parks, gardens, and tree-lined streets, ensuring equitable access to green spaces for all residents, and implementing effective urban planning strategies that integrate green infrastructure into the cityscape.

### 4.3. Urban planning and design strategies for promoting healthy communities

Through a comprehensive evaluation of these aspects, strengths and weaknesses in the existing policies and governance structures of Bangladesh for Dhaka City can be identified. This evaluation can help inform policy reforms and interventions to better address the health risks associated with unplanned urbanization. It is important to ensure that policies are evidence-based, meet population needs, and are implemented in a coordinated manner with the active participation of stakeholders. It is estimated that more than 50 % of the world’s population currently lives in urban areas (11). Evaluating the existing policies and governance structures of Bangladesh for Dhaka City concerning the study on unplanned urbanization and health risks is crucial to understanding the effectiveness of current measures and identifying areas for improvement.

Assessing the urban planning policies in place is essential to determine how effectively they address the challenges of unplanned urbanization and the associated health risks. This includes examining the regulations and guidelines for land use, infrastructure development, and slum upgrading initiatives governance (8). It is important to evaluate policies related to housing and sanitation because they directly affect people’s living conditions and health. This involves assessing the adequacy of policies for the governance of affordable housing, access to clean water, adequate sanitation, and waste management systems (16). It is important to analyze health care policies related to urban slums and vulnerable groups. It involves the assessment of the availability, accessibility, and affordability of health care in Dhaka city, especially in slum areas. Evaluating the extent to which existing policies address the specific health risks faced by slum dwellers is important. Assessing the effectiveness of environmental policies is necessary as the urban environment significantly influences public health. This includes evaluating policies related to air pollution control, green space provision, and environmental sustainability. Analyzing how these policies address the specific environmental determinants of health risks in Dhaka City is crucial.

Evaluating the governance structures and institutional mechanisms is important to understand the coordination and effectiveness of policy implementation. This involves assessing the roles and responsibilities of relevant government departments, agencies, and local authorities in addressing the health risks of unplanned urbanization governance (9, 30). Evaluating the extent of stakeholder engagement in policy development and implementation is essential. This includes analyzing the involvement of community representatives, non-governmental organizations, and other stakeholders in decision-making processes. Assessing the mechanisms for gathering feedback and incorporating community perspectives is important for ensuring inclusive and responsive governance. Evaluating the effectiveness of policy implementation and enforcement is crucial for determining the impact of existing policies on the ground. This involves examining the mechanisms for monitoring and evaluating policy outcomes, as well as the enforcement measures in place to ensure compliance governance (34).

### 4.4. Social networks and community engagement in promoting health

Social networks and community engagement play a crucial role in promoting urban public health in cities like Dhaka. Dhaka, being a densely populated city with numerous health challenges, requires active involvement and collaboration between various stakeholders, including individuals, communities, government agencies, and non-governmental organizations (NGOs). Social networks provide a platform for disseminating important health information to a wide audience. Health organizations and government agencies can use social media platforms like Facebook, Twitter, and Instagram to share public health messages, updates on disease outbreaks, preventive measures, and health promotion campaigns. These platforms help reach a large number of people quickly and effectively.

Social networks enable individuals to connect and form communities based on shared health interests or concerns. Online support groups and communities can provide emotional support, share experiences, and offer guidance to individuals facing health challenges. These networks create a sense of belonging and reduce feelings of isolation, especially for individuals with chronic diseases or mental health conditions. Social networks can serve as a valuable source of health-related data (15). Through online surveys or community forums, public health researchers and organizations can gather data on health behaviors, disease prevalence, and environmental factors. This data can be used to identify public health priorities, plan interventions, and monitor the effectiveness of health programs.

Social networks and community engagement can help mobilize resources and volunteers for public health initiatives. Online platforms can be used to coordinate blood donation drives, health camps, and fundraising campaigns for underprivileged communities (38). The power of social networks lies in their ability to connect people and facilitate collective action toward improving public health. Social networks provide a channel for individuals and communities to engage with policymakers and advocate for better public health policies. Online campaigns and petitions can raise awareness about specific health issues and demand policy changes (34, 44).

Though urbanization is not a negative phenomenon, urban health problems persist in the cities of developing nations as a result of urbanization. Unplanned urbanization poses dangers of profound social instability, as well as threats to infrastructure, environmental, and health weaknesses. In the urban core of developing nations, carbon monoxide emissions have been identified as significant environmental pollutants and health disasters (29). The government should also work with relevant stakeholders to ensure the construction of bypass roads outside of these cores’ grid locations. The autoregression analysis reveals that the location, CO concentration, and other factors are significant determinants of the resident’s daily headache symptoms. This demonstrates the vulnerability of the nucleus (32, 39).

## 5. Implications for future research directions

The findings of this research hold significant implications for future research directions in the field of urbanization and public health, particularly in the context of rapidly growing cities like Dhaka. Firstly, there is a need for further investigation through longitudinal studies into the long-term health impacts of unplanned urbanization. Secondly, research should focus on the interplay between urban planning and health outcomes. Examining how urban policies, such as housing regulations, transportation planning, and green space development, influence public health can guide urban development strategies that prioritize well-being. Thirdly, the role of community-based interventions and community engagement in mitigating health risks should be explored. Fourthly, comparative research with other cities facing similar urbanization challenges can provide valuable insights and policy lessons. Understanding both commonalities and differences in health risks and interventions across cities can inform tailored approaches for Dhaka and similar urban areas globally. Lastly, an interdisciplinary approach is essential for comprehensive research. Collaboration between public health experts, urban planners, economists, sociologists, and policymakers can yield holistic insights into the complex relationships between urbanization and health. These collaborative efforts can result in evidence-based policies and interventions that promote public health in the face of rapid urbanization.

## 6. Strengths and limitations

The strength of this study lies in its comprehensive examination of the multifaceted relationship between unplanned urbanization and public health in Dhaka City. It utilizes a diverse range of data sources, including environmental, demographic, and health-related data, allowing for a holistic examination of the urban environment’s impact on public health. Additionally, the study’s findings contribute valuable knowledge to urban planning and policymaking, providing insights that can inform strategies to mitigate health risks associated with rapid urbanization in not only Dhaka but also similar urban areas globally. However, the limitation of this study is the reliance on secondary data sources, which may have inherent inaccuracies or inconsistencies. It may not account for long-term health impacts. The study’s focus on Dhaka City might not fully capture the diversity of health risks in different urban settings within Bangladesh. Finally, the study does not delve deeply into specific health interventions or policy recommendations. Despite these limitations, it provides valuable insights into the complex associations between urbanization and public health in Dhaka.

## 7. Conclusion

There is an urgent need for better disentangling of the various phenomena associating cities with arbovirus transmission (36) since future estimates show an ever-increasing share of urban populations. Dhaka City in Bangladesh has experienced rapid, uncontrolled urbanization, which has exposed its residents to many of the dangers that come with city living. Through a descriptive, analytical, and exploratory examination of the associations between unplanned urbanization and public health, this study sheds light on the pressing issues and implications for the well-being of the population in Dhaka City. The findings reveal that the rapid and unplanned growth of the city has led to a range of health risks. Socioeconomic disparities, driven by unequal distribution of resources, have created pockets of poverty and marginalization, exacerbated vulnerability, and hindered access to healthcare, education, and basic amenities.

However, the influx of migrants, coupled with population growth, has strained the urban infrastructure, resulting in inadequate housing, overcrowding, and inadequate sanitation, all of which contribute to increased health risks (20). The impacts of these determinants on public health are multifaceted. The prevalence of infectious diseases, such as waterborne illnesses, respiratory infections, and vector-borne diseases, is higher in densely populated urban areas, particularly in slums (22) and informal settlements. Furthermore, the urban environment, characterized by pollution, inadequate sanitation, and limited access to green spaces, poses additional health hazard zones (12). The consequences of these health risks are borne disproportionately by vulnerable populations, including the urban poor, migrants, and marginalized communities (7, 13, 17).

The policy implications and interventions discussed in this study provide a roadmap for addressing the challenges posed by unplanned urbanization and mitigating the associated health risks. Implementing comprehensive urban planning strategies, improving infrastructure development, controlling air pollution, ensuring access to healthcare services, upgrading slums, and promoting public awareness and education are key steps toward creating a healthier and more sustainable urban environment. However, it is important to recognize that addressing the health risks of unplanned urbanization requires a multi-sectoral and holistic approach. Effective governance, intersectoral collaboration, and community participation are crucial for the successful implementation of policies and interventions. Stakeholders from government agencies, urban planners, healthcare providers, and community representatives must work together to integrate health considerations into urban development plans and policies.

The study’s findings underscore the undeniable links between unplanned urbanization and various health risks in Dhaka City. It highlights the pressing need for immediate action in several key areas. Firstly, sustainable urban planning is crucial to guide the city’s growth in a more structured and organized manner, reducing the adverse health impacts associated with haphazard expansion. Secondly, improving infrastructure, particularly sanitation systems, waste management, and access to clean water, is essential to prevent the spread of waterborne diseases and reduce environmental pollution. Thirdly, ensuring equitable access to healthcare services across all socioeconomic groups is imperative. This can help bridge health disparities and provide timely medical attention to those in need. Lastly, targeted interventions should be implemented to address specific public health vulnerabilities arising from unplanned urbanization. These might include initiatives to control vector-borne diseases, improve mental health support, and enhance community resilience. To achieve these goals, a comprehensive and collaborative approach involving urban planners, policymakers, healthcare providers, and community stakeholders is necessary. By working together, Dhaka City can move toward a healthier, more resilient, and inclusive urban environment that prioritizes the well-being of its residents.

## Author contributions

MAR: Conceptualization, Data curation, Formal analysis, Investigation, Methodology, Project administration, Resources, Software, Supervision, Validation, Writing – original draft, Writing – review & editing. AK: Formal analysis, Investigation, Validation, Writing – review & editing. MA-M: Conceptualization, Data curation, Formal analysis, Methodology, Resources, Validation, Writing – review & editing.
